# X-ray microdiffraction analysis of radiation-induced defects in single grains of polycrystalline Fe

**DOI:** 10.1107/S0909049509052078

**Published:** 2010-01-16

**Authors:** E. D. Specht, F. J. Walker, Wenjun Liu

**Affiliations:** a Materials Science and Technology Division, Oak Ridge National Laboratory, Oak Ridge, TN 37831-6118, USA; b Department of Applied Physics, Yale University, New Haven, CT 06520, USA; c Advanced Photon Source, Argonne National Laboratory, Argonne, IL 60439, USA

**Keywords:** microdiffraction, irradiation, diffuse scattering

## Abstract

Single-crystal diffuse X-ray scattering was used to characterize radiation-induced defects in individual grains of a polycrystalline proton-irradiated Fe foil. The grains were probed with an intense 1 µm X-ray beam to demonstrate that both polycrystalline and micrometer-scale samples can be studied with single-crystal-like signal-to-noise. Scattering was measured with an X-ray-sensitive area detector, which measures intensity over a surface in reciprocal space. By scanning the X-ray energy, the intensity was measured over reciprocal-space volumes. Since the sample is not rotated, the real-space scattering volume does not change. Methods to minimize experimental artifacts arising from the use of an area detector are described.

## Abstract 2

Single-crystal diffuse X-ray microdiffraction was used to characterize radiation-induced defects in individual grains of a polycrystalline proton-irradiated Fe foil.

## 1. Introduction   

The effects of irradiation of fuel and structural materials in fission and fusion reactors must be understood and controlled to avoid deleterious swelling, hardening and embrittlement. Primary damage typically consists of interstitials and vacancies which diffuse to form clusters and more extended defects such as voids, bubbles and dislocation loops (Gittus, 1978 ▶). Measurement of the structure of such lattice defects uses two complementary methods: transmission electron microscopy (TEM) is used to examine individual extended defects; X-ray and neutron diffraction provide statistical information about the population of defects. While TEM isolates the defect with high direct-space (spatial) resolution, diffraction uses reciprocal-space (angular) resolution. Diffraction from the undisturbed lattice is concentrated in sharp Bragg peaks at discrete reciprocal lattice vectors, while defects redistribute the scattering more diffusely in reciprocal space. The location, shape and symmetry of the diffuse scattering is used to determine the type of defect, while the intensity of the scattering depends on both defect density and defect type (Dederich, 1971 ▶).

Diffraction analysis of radiation-induced lattice defects is best performed using single-crystal samples (Ehrhart, 1994 ▶). The diffuse scattering from defects is weak even from single crystals and powder averaging from a polycrystalline sample further smears the weak scattering. Worse yet, information about the shape of the diffuse scattering is lost in powder averaging. As a result, polycrystalline samples have until now been suitable only for extracting the most rudimentary information about even highly defective materials.

Unfortunately, single crystals are not readily available for many technically important alloys. Here we demonstrate how synchrotron X-ray microdiffraction can be used to analyze lattice defects in a polycrystalline irradiated material with single-crystal-like sensitivity. By focusing a bright synchrotron beam onto a single grain, elastic strain, grain orientation (Ice *et al.*, 2005 ▶) and plastic deformation (Barabash *et al.*, 2003 ▶) can be measured by treating the grain as a small single crystal.

A further advantage of this technique is that it can be applied to very small samples, in principle down to ∼10 µm in size. Even highly radioactive materials such as spent reactor fuel can be handled with minimal restrictions for such sizes, and the background signal from sample radiation is reduced as the sample is made smaller. For reasons of convenience, these demonstration measurements used a proton-irradiated sample which was not radioactive.

Once the beam is focused to a volume less than the grain size, the resulting diffraction can be analyzed almost as if it were from a single crystal. A significant exception is that conventional diffuse scattering measurements on single crystals are made by rotating the sample under illumination with a monochromatic beam of fixed energy. Because sample rotation changes the illuminated volume, different grains of a polycrystalline material will contribute to the diffraction as the sample rotates. Therefore, microdiffraction reciprocal-space maps are made by scanning the energy of a monochromatic beam while holding the sample orientation fixed (Ice *et al.*, 2005 ▶). An area detector, rather than the point detector used in traditional measurements, is used to measure diffracted intensity, which accelerates data collection. We will show, however, that the use of an area detector introduces artifacts and will discuss how this limitation may be overcome.

## 2. Experimental   

A 10 mm × 10 mm × 0.1 mm 99.99% Fe (metals basis, Alfa Aesar) rolled foil was annealed at 1073 K in a mixture of 4% H_2_ in Ar, yielding a typical body-centered cubic recrystallization texture (Barrett & Massalski, 1980 ▶) with a grain size of ∼50 µm. The sample was irradiated with 2.5 MeV protons at a rate of 2 × 10^17^ m^−2^ s^−1^ with total fluence 8 × 10^20^ m^−2^. The temperature on the surface, monitored with an infrared thermal imaging camera, was kept below ∼323 K. One half of the sample was masked so as to remain unirradiated.

Microdiffraction measurements were made at beamline 34-ID-E of the Advanced Photon Source, Argonne, IL, USA (Ice *et al.*, 2005 ▶). Undulator radiation was focused to ∼1 µm^2^ using Kirkpatrick–Baez mirrors and energy-filtered with a double-crystal Si(111) monochromator. A three-axis translation stage was used to scan the sample position, while sample orientation was kept fixed with the beam incident at a 45° angle. The sample was cooled to 77 K using a Joule–Thomson refrigerator powered by compressed N_2_ gas. Diffracted X-rays were imaged with a 2048 × 2048 pixel, 50 mm × 50 mm, cooled, 16-bit charge-coupled device (CCD) detector (Roper Scientific PI SCX 4300), shielded from the sample’s Fe *K* fluorescence by a 0.175 mm-thick Al filter. A reflection geometry was used, with a scattering angle near 90°. The sample–detector distance was 60 mm.

Detector counts were normalized to an air-filled ionization chamber monitoring incident X-ray flux. To compensate for the energy-dependent efficiency of the ion chamber, the ionization current was divided by the nitrogen photoelectric cross section and by the X-ray energy. A correction was made for absorption by the Fe filter. Cross sections were taken from McMaster *et al.* (1969 ▶). The absolute normalization to electron units is made by measuring thermal diffuse scattering around the (400) reflection of unirradiated Fe at room temperature. At a reciprocal lattice vector **Q** = (*Q*, 0, 0) close to a Bragg reflection **G** = (*G*, 0, 0), the dominant term is first-order temperature diffuse scattering: in electron units (Warren, 1990 ▶),
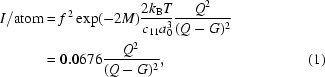
where *f* = 8.59 is the atomic form factor (Prince, 2004 ▶), *M* = 0.236 is the Debye–Waller factor (Paakkari, 1974 ▶), *k*_B_ = 1.381 × 10^−23^ J K^−1^ is Boltzmann’s constant, *T* = 298 K is the temperature, *c*_11_ = 2.331 × 10^11^ Pa is the elastic constant (Rayne & Chandrasekhar, 1961 ▶) and *a*_0_ = 0.2886 nm is the lattice parameter (von Batchelder & Raeuchle, 1954 ▶).

Conventional X-ray diffuse scattering measurements are typically made by cutting one or more single crystals with flat surfaces which give access to the desired regions of reciprocal space. Polycrystalline samples have the advantage of providing a variety of crystal orientations, but the experimenter must locate the right one. To study, for example, diffuse scattering around a (330) Bragg reflection, the incident energy was first tuned to 13.50 keV, corresponding to the Bragg angle θ = 45° at the detector’s center for this reflection. Next, the sample position was scanned in small increments while watching for scattering near the detector’s center. While the probability that we will find a crystal grain so precisely oriented as to excite a strong Bragg reflection is vanishingly small, we readily found grains with orientations close enough to (330) to generate diffuse scattering clearly visible in a 1 s exposure. These measurements were made at room temperature, so that even unirradiated samples generated detectable thermal diffuse scattering. Once a properly oriented grain was located, a diffraction image was taken using a polychromatic beam; this image was indexed to precisely determine the grain orientation (Chung & Ice, 1999 ▶), which was used to tune the monochromator to other points in reciprocal space as described in Appendix *A* [App appa].

In this work, the beam size and X-ray penetration depth are much smaller than the grain size, so diffraction is observed from a single grain, as shown by the polychromatic beam (*i.e.* Laue) diffraction pattern. The diffraction can thus be analyzed as if it were from a single crystal. For measurements of finer-grained samples, where multiple grains are illuminated, the polychromatic beam diffraction pattern will have to be analyzed to insure that scattering from neighboring grains does not overlap the reciprocal-space region of interest.

Data were acquired as a set of CCD diffraction patterns at energies around the Bragg energy, the energy at which the grain satisfies the Bragg condition for the selected reciprocal lattice point. A series of energy scans was used to measure both strong rapidly varying scattering near the Bragg peak and weak slowly varying scattering far from the Bragg peak, with exposures ranging from 0.1 to 1500 s and energy steps ranging from 10 to 100 eV.

The strong Bragg reflections caused two instrumental artifacts. First, an afterglow appears on the CCD after exposure to the Bragg reflection: a residual signal appeared on several heavily exposed pixels for about an hour after the exposure. Second, the detector’s point spread function has a large effect on the diffraction pattern. As will be seen when our results are presented, instrumental broadening in the CCD causes scattered intensity from the Bragg peak to spill over into neighboring pixels. A beamstop was used to minimize these artifacts. Since the position of the Bragg reflection varies with grain orientation, the beam stop must be mobile to work with various grains. A Pt wire, 50 µm in diameter, was used in some of the measurements to block the center of the Bragg reflection.

## 3. Results   

**Figure 1:**
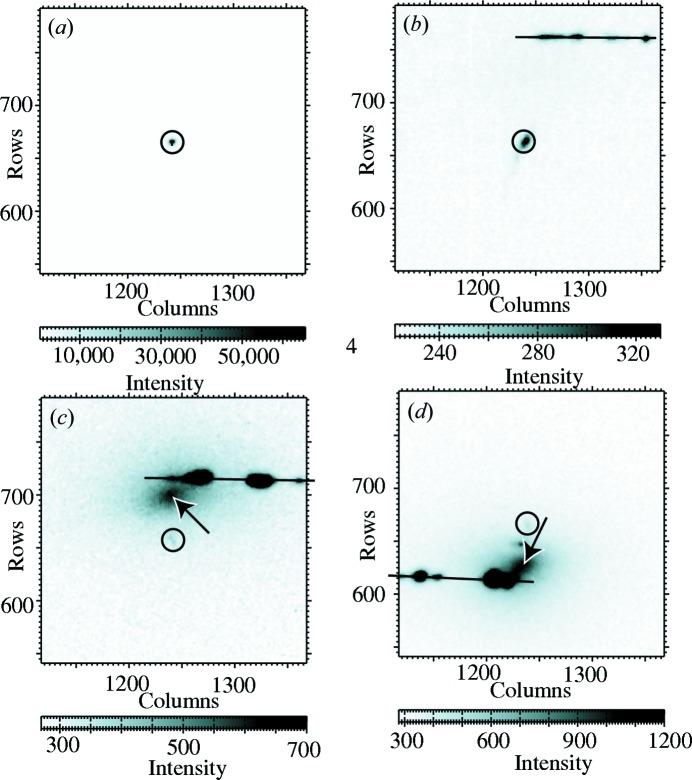
Detector images for diffraction from proton-irradiated Fe at 77 K, near the (033) reflection, with data collection parameters given in Table 1 ▶. The bars below images indicate rescaling of the images to show weak features. Panel (*a*) shows the Bragg reflection at the Bragg energy. The circles indicate the same detector position on the other images, where an afterglow is visible (*i.e.* a detector artifact). Lines indicate Debye rings owing to crystal mosaicity. Arrows indicate diffuse scattering. (See Fig. 2 ▶ for the scattering geometry for these features.)

**Figure 2:**
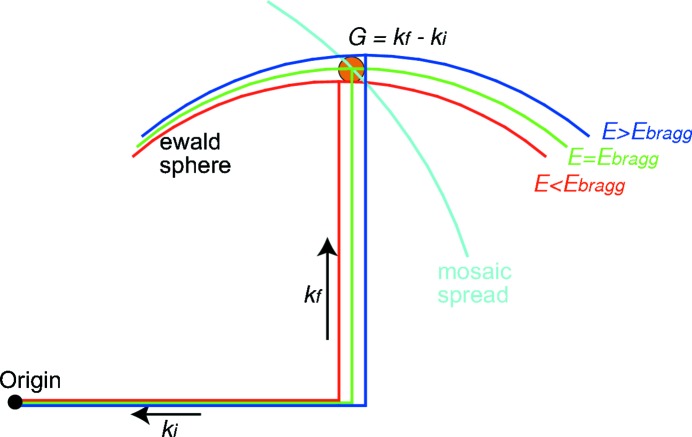
One reciprocal-space plane, illustrating the scattering geometry. Each color denotes one X-ray energy, for which the area detector measures scattering on a Ewald sphere centered on the tail of the diffracted wavevector *k*_*f*_; *k*_*i*_ is the incident wavevector. Scattering into the detector’s center, corresponding to the head of *k*_*f*_, shifts radially in reciprocal space as energy changes. The circle represents a sphere of diffuse scattering, with a Bragg reflection *G* at its center. Bragg scattering from misoriented portions of the sample falls on a sphere of mosaic spread, centered on the origin of reciprocal space. The intersection of the mosaic spread with the Ewald sphere corresponds to an arc on the detector which is close to a horizontal line (Fig. 1 ▶). For *E* = *E*_Bragg_, both diffuse scattering and mosaic spread are centered on the Bragg reflection. For *E* < *E*_Bragg_, diffuse scattering is at higher scattering angles than observed for the Bragg reflection at the Bragg energy, with mosaic spread at still higher angles [Fig. 1(*c*) ▶]. For *E* > *E*_Bragg_, diffuse scattering is at lower angles, with mosaic spread at even lower angles [Fig. 1(*d*) ▶].

**Figure 3:**
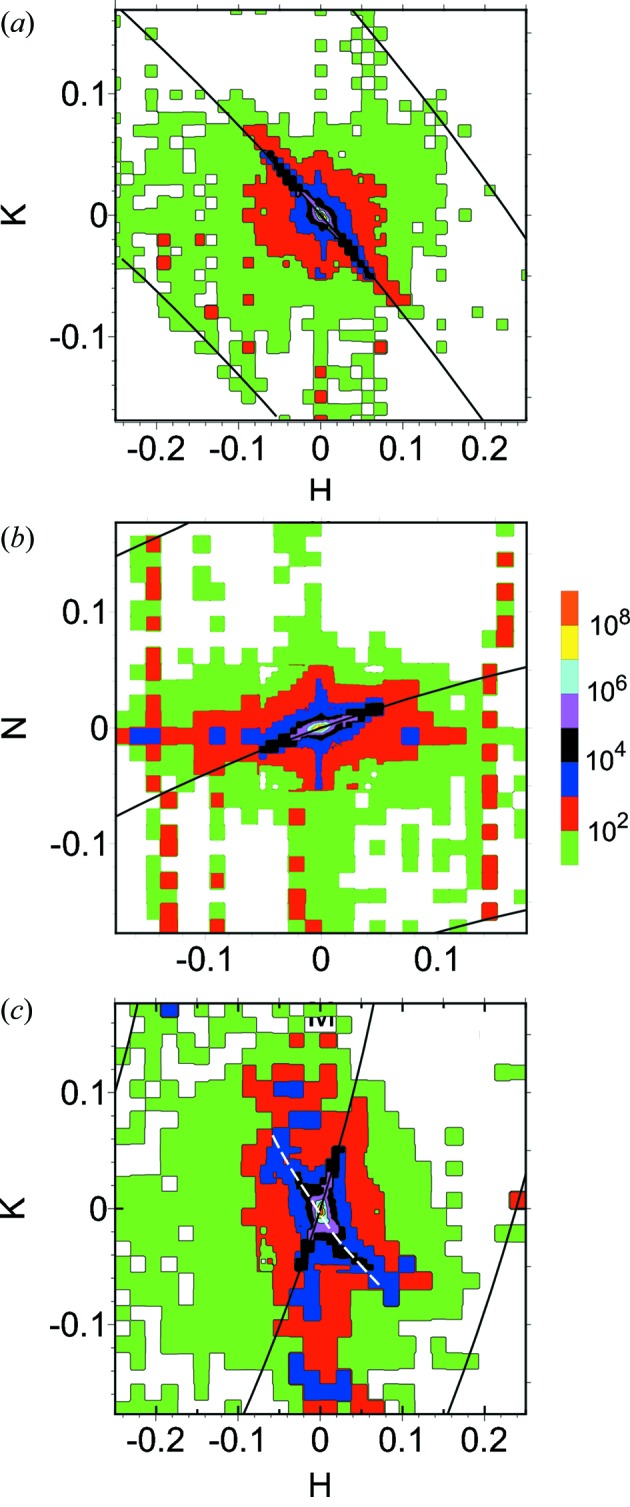
Reciprocal-space sections of scattering from proton-irradiated Fe at 77 K: (*a*) (*H*, 3 + *K*, 3 + *K*), (*b*) (0, 3 + *M* + *N*, 3 − *M* + *N*), (*c*) (*H*, 3 + *K*, 3 − *K*). Black lines indicate minimum and maximum measured energies and the energy at the central Bragg reflection. The white dashed line indicates the direction of crystal deformation. The vertical streak in panels (*a*) and (*b*) is due to afterglow in the detector from the strong Bragg reflection. The contour lines and color bar on the right separate factor of 10 changes in intensity.

Diffuse scattering, indicated by arrows in Figs. 1(*c*) and 1(*d*) ▶ ▶, is apparent in the raw CCD images. However, a straightforward analysis is complicated by the fact that the weak diffuse scattering is actually close in reciprocal space to very intense scattering, as illustrated in Fig. 2 ▶. For example, when the scattering in reciprocal-space sections is plotted in Fig. 3 ▶, it can be seen that the diffuse scattering is dominated by three instrumental artifacts. A streak of strong scattering is apparent in each two-dimensional section, corresponding to the intersection with that section and the Ewald sphere corresponding to the Bragg energy. In three dimensions, there is a spherical shell of scattering. This direction corresponds to spreading of the Bragg reflection, presumably in the CCD. Scattering in the monochromator or mirrors leads to characteristic streaking in other reciprocal-space directions. The beam spreading is similar for irradiated and unirradiated samples and similar at room temperature and 77 K, ruling out diffuse scattering from the sample. Circular symmetry about the Bragg reflection is apparent in the CCD images.

**Figure 4:**
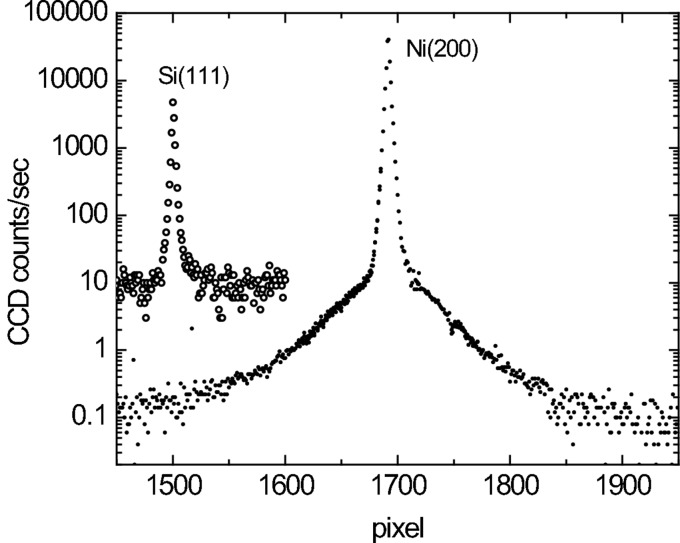
One column of the CCD image through an irradiated Fe(002) reflection at 77 K, illustrating the point spread function of the detector. To increase the dynamic range of the detector, three images with exposure times of 0.5 s, 10 s and 100 s have been combined. A constant background has been subtracted. Shown for comparison is a column through a (111) reflection from a 15 µm-thick Si wafer.

The spatial resolution or point spread function of the CCD is shown in Fig. 4 ▶, which plots a single column from the CCD. While the FWHM of the scattering profile is only 2.5 pixels, the tails extend far beyond. The scattering falls off rapidly for the first ±20 pixels, corresponding to ±0.02 reciprocal lattice units (RLUs); this is the part of the scattering which falls on a spherical shell and is thus attributed by us to the detector point spread function. The scattering further away from the Bragg reflection, falling off more slowly, is roughly isotropic about the Bragg peak as expected for diffuse scattering from lattice defects. A 15 µm-thick Si wafer produces similar scattering close to the (111) Bragg reflection. Since the Si wafer produces only weak diffuse scattering and negligible broadening owing to beam penetration, this similarity confirms that it is the detector rather than the sample which causes this shell of scattering.

A weaker streak can be seen in Figs. 3(*a*) and 3(*b*) ▶ in the vertical direction, which is parallel to the scattering vector. These reciprocal-space points correspond to the pixels on the CCD at which the Bragg reflection is observed, marked with circles in Fig. 1 ▶. This scattering is strong just after a Bragg reflection is excited, decaying gradually with time as an afterglow in the CCD. Diffuse scattering measurements must avoid this reciprocal-space direction as well.

Fig. 3(*c*) ▶, which shows a slice transverse to the scattering vector, illustrates a third artifact. A streak of scattering follows a direction which varies from grain to grain, showing that it is not instrumental in origin. Each point on the streak corresponds to the crystal orientation at a different point in the path of the beam through the grain; the direction of the streak is determined by the deformation tensor of the grain (Barabash *et al.*, 2003 ▶). The intrinsic diffuse scattering profile can be measured in other directions. In the CCD images, this subgrain structure appears as intense spots forming Debye–Scherrer rings, marked with lines in Fig. 1 ▶.

**Figure 5:**
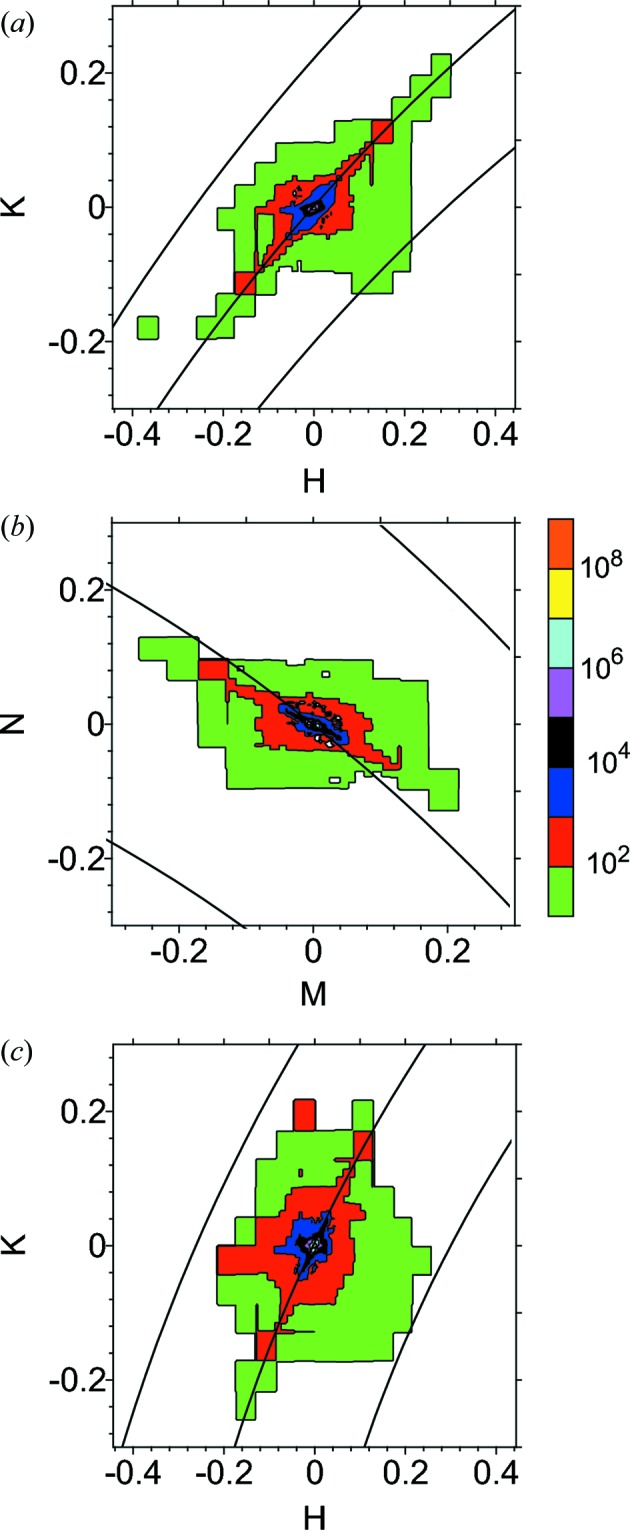
Reciprocal-space sections of scattering from proton-irradiated Fe at 77 K: (*a*) (*H*, 3 + *K*, 3 + *K*), (*b*) (0, 3 + *M* + *N*, 3 − *M* + *N*), (*c*) (*H*, 3 + *K*, 3 − *K*). Lines indicate minimum and maximum measured energies and the energy at the central Bragg reflection. Wire is blocking the central Bragg reflection.

Fig. 5 ▶ shows reciprocal-space sections similar to those shown in Fig. 3 ▶ but with a wire blocking the central Bragg reflection. Because a different grain was illuminated, the mosaic spread and Ewald sphere sections are in different directions than in Fig. 3 ▶. Artifacts owing to spreading and afterglow of the Bragg reflection in the CCD are minimized so the intrinsic scattering can be more clearly measured. Alternatively, Bragg reflections can be blocked using movable magnets placed on the face of the area detector (Fábry *et al.*, 2006 ▶).

**Figure 6:**
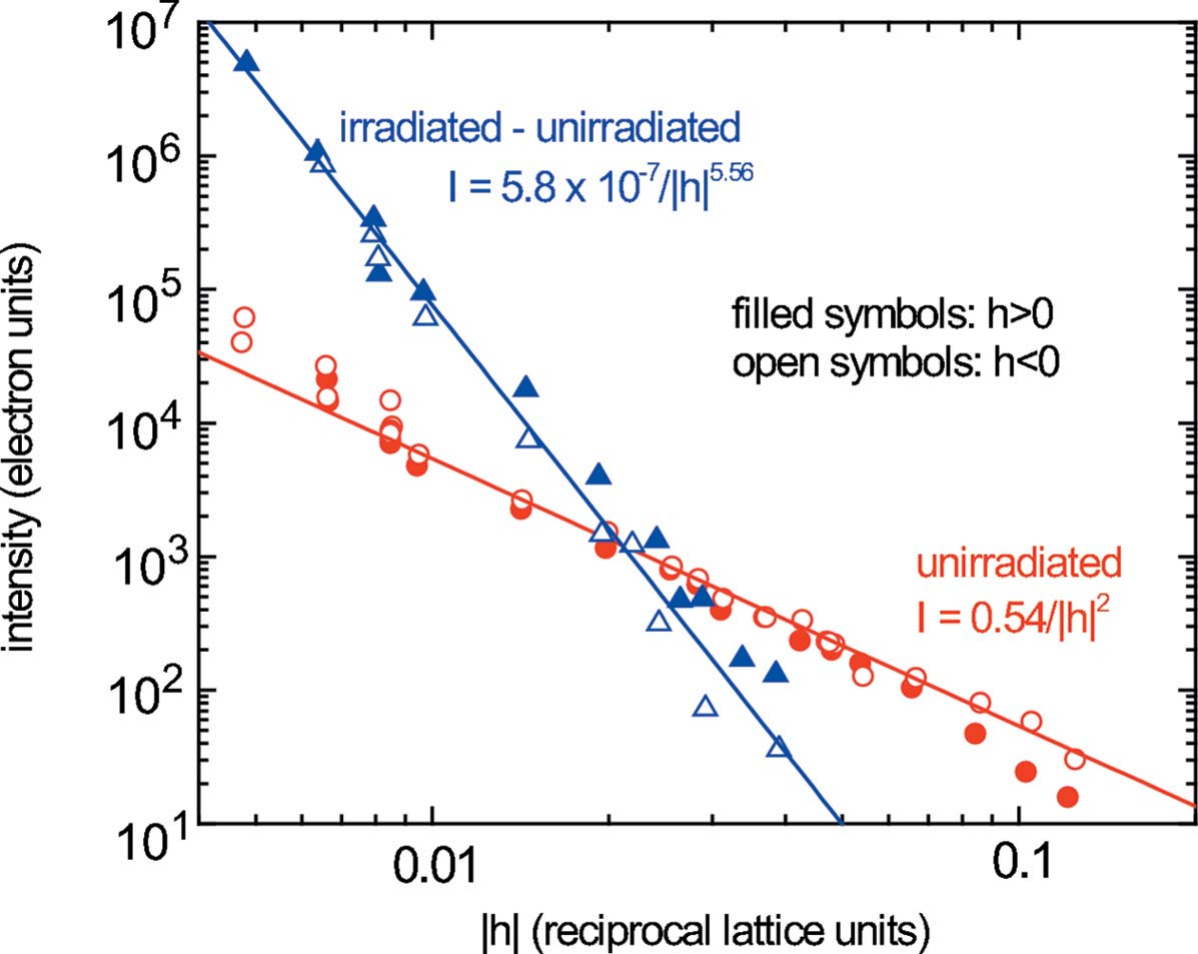
Excess scattering from proton-irradiated (triangles) and total scattering from unirradiated (circles) Fe at 77 K along (4 + 0.86*h*, 0.5*h*, 0).

**Figure 7:**
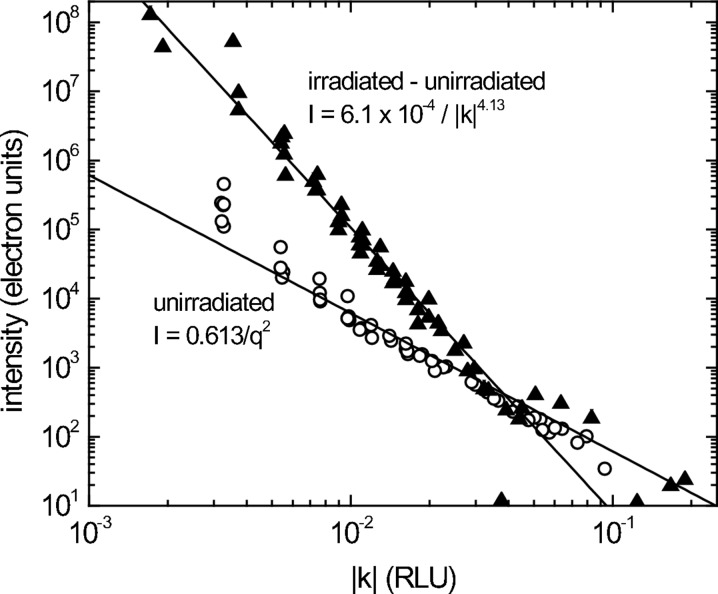
Excess scattering from proton-irradiated (triangles) and total scattering from unirradiated (circles) Fe at 77 K along (4*k*0).

While a beam stop blocking the central Bragg reflection is needed to measure two-dimensional sections of reciprocal space, linear reciprocal-space profiles can be measured by choosing directions which avoid instrumental artifacts. Shown in Figs. 6 ▶ and 7 ▶ are intensity profiles in the [0.86, 0.5, 0.0] and [010] direction through a (400) reflection; none of the three observed artifacts occurs along these lines, so the intrinsic scattering profile is observed. An unirradiated sample at 77 K gives the *I* ≃ 1/*q* ^2^ (where *q* = *Q* − *G*) profile expected for first-order thermal diffuse scattering (Figs. 6 ▶ and 7 ▶, circles) (Warren, 1990 ▶). Subtracting this thermal scattering from the scattering from the proton-irradiated sample leaves scattered intensity owing to defects which fall off as *I* ≃ 1/*q* ^5.56^ (Fig. 6 ▶, triangles) and *I* ≃ *q* ^4.13^ (Fig. 7 ▶, triangles).

The scattering is symmetric within experimental uncertainty, *i.e.* *I*(*q*) = *I*(−*q*). The experimental uncertainty at small *q* is due largely to incidental problems in monitoring incident beam intensity. Uncertainty at larger *q* arises mainly from subtracting the background scattering owing to Compton scattering and sample fluorescence.

## 4. Discussion   

The data contain strong instrumental artifacts. Can we be confident that we have properly measured diffuse scattering from the sample? The defect-induced scattering is analyzed by measuring the difference in scattering between an irradiated and an unirradiated sample, so the signal we measure must be caused by defects. The thermal scattering measured from an unirradiated Fe grain at room temperature closely follows the expected *I* ≃ 1/*q* ^2^ dependence, so instrumental distortion of scattering is apparently not significant.

**Figure 8:**
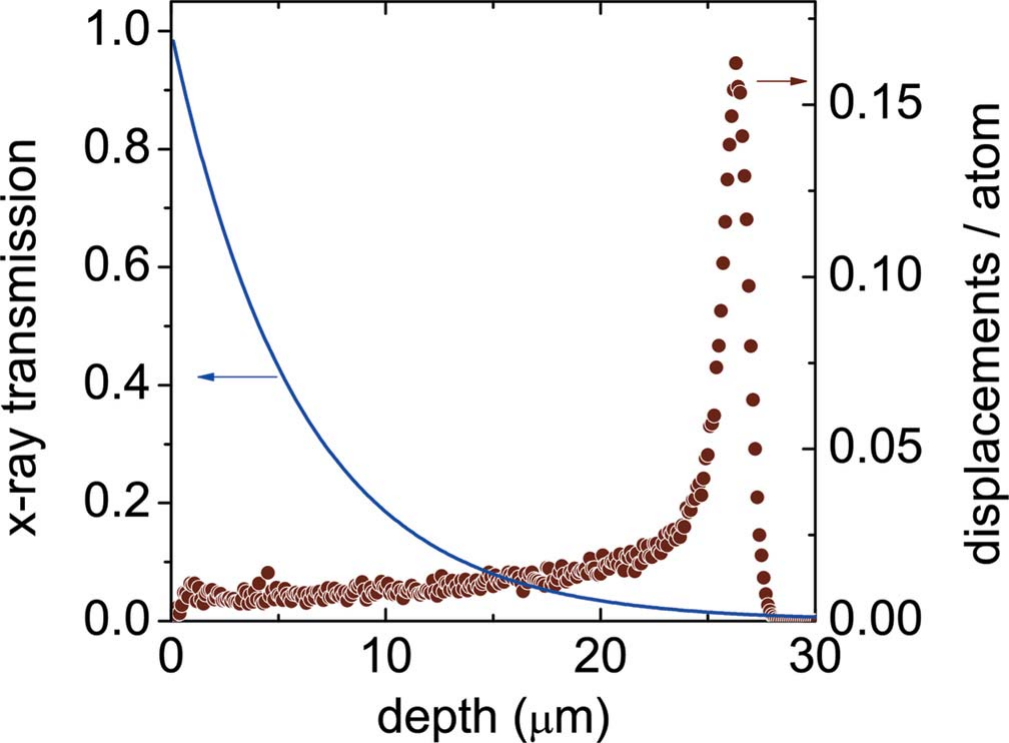
Depth dependence of dose for 2.5 MeV protons (circles) and transmission of diffracted 13.5 keV X-rays (line) in Fe.

Ion irradiation does not affect the sample uniformly, so X-ray diffraction will sample a range of doses. As shown in Fig. 8 ▶, ion dose increases from 0.064 displacements per atom (dpa) near the surface to a peak of 0.16 dpa at a depth of 26 µm. Ion range was calculated using the *SRIM-2000* code (Ziegler *et al.*, 2008 ▶). The effect of the high-dose layer is reduced by the absorption of X-rays. The penetration depth of 13.5 keV X-rays in Fe is 16.8 µm (McMaster *et al.*, 1969 ▶). For diffraction with incident and diffracted rays at a 45° angle, the effective penetration depth is *p* = 5.9 µm. Weighting the dose σ at depth *t* by the transmission, the average dose sampled by the diffracting X-rays is 〈σ〉 = ∫d*t*exp(−*t*/*p*)σ(*t*)/*p* = 0.084 dpa. The high-dose layer will have a significant effect on the diffraction pattern only if large clusters form, since these cause disproportionately strong scattering.

Scattering from defects of size *S* is generally dominated by long-range displacements for *qS* << 1, producing Huang scattering which scales as *I* ≃ 1/*q* ^2^. For *qS* >> 1, scattering comes mainly from defect cores and first falls off as *I* ≃ 1/*q* ^4^ (the Stokes–Wilson region), and then falls off increasingly steeply with increasing *q* (Dederich, 1973 ▶).

Scattering from defects in the proton-irradiated Fe sample scales as *I* ≃ 1/*q* ^5.56^ (longitudinal) and *I* ≃ 1/*q* ^4.13^ (transverse) over the entire measured range, 0.1 nm^−1^ < *q* < 1.0 nm^−1^, where *q* = 2π*h*/*a*_0_. Thus, the measurements fall well into the Stokes–Wilson region, so the defects must be greater than ∼10 nm in size. Of course, it is speculative to base conclusions on a cross-over which has not been observed; measurements at lower *q* will provide clearer evidence of defect size. Conventional diffuse X-ray scattering measurements of neutron-irradiated Fe find interstitial defect clusters which are ∼1.4 nm in size (Stoller *et al.*, 2007 ▶). It is not clear why proton irradiation leads to clusters so much larger in size.

Additional characterization of these large clusters will require measurements to smaller *q*. The measurements described above were limited by the instrumentation of beamline 34-ID-E at the Advanced Photon Source, which uses an X-ray beam with divergence Δθ = 1 mrad. The resolution at wavelength λ and Bragg angle θ is Δ*q* = 4πcosθΔθ/λ for θ = 45° and λ = 0.075 nm, Δ*q* = 0.1 nm^−1^.

Fortunately, scattered intensity at small *q* is large, so the diffuse scattering signal will be sufficient even with reduced Δθ. Resolution may be increased up to the diffraction limit Δθ = 1.2λ/*R*, where *R* is the focal beam size. At this limit, Δ*qS* = 15*S*cosθ/*R*. We must have *R* >> *S* to obtain a statistical sampling of defects, so a resolution-limited beam will always provide sufficient resolution to resolve the Huang scattering region *qS* << 1. Elliptical X-ray mirrors have been fabricated with r.m.s. figure errors as small as ∼0.5 µrad (Liu *et al.*, 2005 ▶) corresponding to a resolution of Δ*q* ≃ 2 × 10^−4^ nm^−1^ and a defect size of *S* ≃ 5 µm.

Correspondingly high angular resolution of the diffracted beam requires an increase in the distance between sample and detector or a decrease in the detector pixel size. For a detector pixel size *p* = 10 µm, a resolution of Δ*q* = 0.001 nm^−1^ is obtained for a sample–detector distance *D* = 4π*p*cosθ/(λΔ*q*) = 1.2 m. A detector at this distance will span only a small solid angle, so a second detector, placed closer to the sample, would be needed to determine grain orientation. It would be difficult to find a grain which diffracts into the distant detector, so either the sample would be rotated to steer the diffracted beam into that detector or the detector would be moved to intercept the diffracted beam. Moving the detector is a more feasible option for very small incident beams: unless the diffracting grain is precisely on the center of rotation, rotating the sample will move the grain out of the beam.

Analysis of smaller defects will require extending data to higher *q*. For this experiment, high-*q* measurements are limited by background thermal scattering, so cooling to lower temperatures would be required. Once thermal scattering is reduced, the remaining limitation will be background from Compton scattering and X-ray fluorescence. In a conventional diffuse-scattering measurement using a point detector, this background can be eliminated by using a wavelength-dispersive monochromator or energy-discriminating detector. When using an area detector, X-ray fluorescence can be removed by an absorbing filter, where the incident X-ray energy is set high enough to pass through the filter with minimal attenuation. The energy loss in Compton scattering is too small to apply this technique, but recently developed pixel-array detectors can provide the energy discrimination needed to remove Compton scattering (Broennimann *et al.*, 2006 ▶).

## 5. Conclusions   

X-ray microdiffraction is used to obtain single-crystal diffuse scattering maps from individual grains in polycrystalline materials, demonstrating that defects can be characterized in micrometer-sized samples. The scattering volume is kept fixed by scanning energy rather than rotating the sample. An area detector is used to create reciprocal-space maps in three dimensions in the time required to obtain one-dimensional scattering profiles with a point detector. This technique will enable the study of highly radioactive samples with minimal radiation exposure.

Several issues arise from the use of an area detector which has no collimation or energy discrimination. Background levels from Compton scattering and X-ray fluorescence are high, so measurements to high *q* will require energy filtration and advanced energy-discriminating detectors. The strong Bragg reflection causes artifacts as it spreads spatially and persists in time in the detector. These artifacts can be minimized by using a beam-stop to mask the central Bragg reflection. Measurements at low *q* will require both better-collimated incident beams and either a larger sample–detector distance or an area detector with finer spatial resolution.

## Figures and Tables

**Table 1:** Parameters for images in Fig. 1 ▶

Panel	Energy (keV)	Exposure duration (s)	Exposure start time (s)
(*a*)	14.327	0.5	0
(*b*)	14.027	100	562
(*c*)	14.169	1500	15897
(*d*)	14.485	1500	20484

## Appendix A Crystallographic computations

Conventional diffuse scattering measurements scan reciprocal space by rotating a sample with a fixed incident-beam energy. Here, the orientations of the incident beam and of the sample are held fixed while the incident beam energy is varied to scan reciprocal space. How do we find a location in reciprocal space?

As discussed by Chung & Ice (1999 ▶), the instrument must be precisely calibrated to determine the incident beam direction , the CCD pixel (*c*_*x*_, *c*_*y*_) which is the detector’s optical center, the rotation tensor ***D*** of the detector, and the distance *d* from the sample to the detector center (*c*_*x*_, *c*_*y*_). The orientation matrix ***U*** of the grain’s reciprocal lattice is found from a polychromatic beam diffraction pattern. The lattice parameter matrix ***B*** is found by adding a monochromatic beam energy scan through one Bragg reflection.

Each reciprocal-space vector **h** is converted to a laboratory-frame scattering vector **Q** = (***UB***)^−1^ **h**. From Bragg’s law, the wavelength will be λ =  and the outgoing wavevector **q**_out_ =  in the detector frame. The diffracted spot will appear on the CCD at [*c*_*x*_ + (*Q*_out,*x*_ *d*/*s*_*x*_ *Q*_out,*z*_), *c*_*y*_ + (*Q*_out,*y*_ *d*/*s*_*y*_ *Q*_out,*z*_)], where (*s*_*x*_, *s*_*y*_) is the pixel size.

Conversely, a reciprocal-space vector can be calculated from a CCD position and X-ray wavelength. A diffracted beam which hits the CCD at pixel (*p*_*x*_, *p*_*y*_) is travelling in the direction **v**_out_ = ***D*** ^−1^[(*p*_*x*_ − *c*_*x*_)*s*_*x*_, (*p*_*y*_ − *c*_*y*_)*s*_*y*_, *d*)]. The reciprocal-space vector will be **h** = .
